# Evidence for percolation diffusion of cations and reordering in disordered pyrochlore from accelerated molecular dynamics

**DOI:** 10.1038/s41467-017-00708-z

**Published:** 2017-09-20

**Authors:** Romain Perriot, Blas P. Uberuaga, Richard J. Zamora, Danny Perez, Arthur F. Voter

**Affiliations:** 1 Materials Science and Technology Division, Los Alamos National Laboratory, P.O. Box 1663, Los Alamos, NM 87545 USA; 2 Theoretical Division, Los Alamos National Laboratory, P.O. Box 1663, Los Alamos, NM 87545 USA; 3 Present Address: Theoretical Division, Los Alamos National Laboratory, P.O. Box 1663, Los Alamos, NM 87545 USA

## Abstract

Diffusion in complex oxides is critical to ionic transport, radiation damage evolution, sintering, and aging. In complex oxides such as pyrochlores, anionic diffusion is dramatically affected by cation disorder. However, little is known about how disorder influences cation transport. Here, we report results from classical and accelerated molecular dynamics simulations of vacancy-mediated cation diffusion in Gd_2_Ti_2_O_7_ pyrochlore, on the microsecond timescale. We find that diffusion is slow at low levels of disorder, while higher disorder allows for fast diffusion, which is then accompanied by antisite annihilation and reordering, and thus a slowing of cation transport. Cation diffusivity is therefore not constant, but decreases as the material reorders. We also show that fast cation diffusion is triggered by the formation of a percolation network of antisites. This is in contrast with observations from other complex oxides and disordered media models, suggesting a fundamentally different relation between disorder and mass transport.

## Abstract (web summary)

Diffusion plays an important role in sintering, damage tolerance and transport. Here authors perform classical and accelerated molecular dynamics simulations of vacancy-mediated cation diffusion in Gd_2_Ti_2_O_7_ pyrochlore and report non-monotonic evolution of cation diffusivity.

## Introduction

Complex oxides are critical components of numerous materials applications. Whether as solid oxide fuel cells^1^, supercapacitors^2^, memristors^3^, or matrices for nuclear waste encapsulations^4^, the versatility of structures and chemical compositions of complex oxides has been exploited to maximize desired properties. Mass transport plays a key role for many of these applications, through for instance ionic conductivity and radiation damage evolution. In addition, diffusion dictates sintering^5^ and aging^6^ of complex oxide materials.

Pyrochlores are a class of complex oxides with the formula A_2_B_2_O_7_, where A is usually a rare earth (Gd^3+^, La^3+^, Lu^3+^, etc.) and B a transition metal (Ti^4+^, Mo^4+^, Zr^4+^, etc.). Among the promising properties offered by pyrochlores, amorphization resistance^7, 8^ and fast ion conduction^9, 10^ stand out. The former was linked to the ability of pyrochlores to disorder by forming antisites A_B_ and B_A_ (i.e., an A cation on a B site and vice-versa), and, along with the occurrence of natural uranium-bearing analog^11^, motivates the use of pyrochlores as nuclear waste encapsulation matrices^12–14^. Ionic conductivity, in contrast, arises from the structural characteristics of the material: the pyrochlore structure is related to the parent fluorite (BO_2_) structure, only with distinct A and B sublattices and vacant oxygen sites to account for the reduced valence. While these structural vacancies are immobile or very slow in the ordered material, cation disorder activates these carriers, leading to a tremendous increase of the ionic conduction^15–17^. Experimentally, it was notably found that disordered pyrochlores can show an increase in their ionic conductivity by up to four orders of magnitude in the Gd_2_(Ti_*x*_Zr_1−*x*_)_2_O_7_ system, with the disorder level increasing with the fraction of Zr introduced^18, 19^. As the disorder is the key to fast ion conduction, it is essential to be able to predict the material’s cation evolution to understand ionic conductivity.

Thus, for both these applications, understanding the dynamics of cations is critical. Further, cation diffusion governs processes such as sintering and aging. However, little is known about the dynamics of cations, especially when the material is in a disordered state due to temperature^20, 21^ (sintering), radiation damage (self-irradiation due to immobilized nuclear waste^4, 22^, extrinsic irradiation through ion beam studies^23, 24^), or chemistry (used to induce disorder in studies of ionic conductivity^19, 25^). From a simulation perspective, this is due in part to the difficulty of reaching timescales relevant to cation diffusion while accounting for the much faster oxygen diffusion. For this reason, earlier work focused on the properties of defect complexes^26^ or isolated cation defects^27^ in otherwise ordered material, where oxygen is mostly immobile. Additionally, the role of cation Frenkel pair defects^28^ and cation interstitials^29^ on the radiation tolerance of pyrochlores was investigated by Chartier et al., while Devanathan et al. probed the role of the chemistry^30, 31^.

**Fig. 1:**
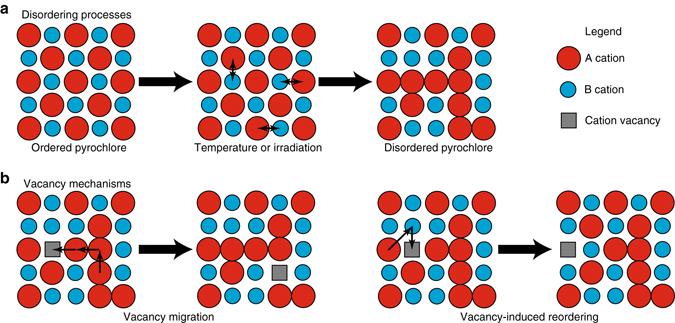
Schematic of the disordering processes and vacancy mechanisms in pyrochlore examined in this manuscript. **a** Disordering processes. **b** Vacancy mechanisms. Oxygen ions omitted for clarity. In **b**, the schematic mechanisms consist of multiple ion moves that, in reality, might be described by multiple saddle points

In the present paper, we used molecular dynamics (MD) and parallel trajectory splicing (ParSplice)^32^, one of the accelerated molecular dynamics methods^33^, to simulate cation vacancy diffusion in disordered Gd_2_Ti_2_O_7_ (GTO) pyrochlore on the microsecond timescale. The disordering processes, which can be caused by, for example, temperature or irradiation, are shown in Fig. 1a, while the types of cation migration and reordering mechanisms of interest are shown in Fig. 1b. In the remainder of the paper, disorder is to be understood as specifically cation disorder, i.e., cation antisites as shown in Fig. 1a. In particular, we are interested in structures in which the cations disorder, but retain crystallinity; we are not examining structurally disordered (or amorphous) structures. We find that a cation vacancy exhibits slow diffusion until a critical level of disorder is reached, at which point fast diffusion is triggered. Along its path, the vacancy can eliminate pairs of antisites, resulting in a concurrent decrease both in the disorder and, consequently, the diffusivity. The migration behavior of the cation vacancy also exhibits signatures of percolation diffusion, such that the relationship between disorder and migration is complex: low disorder does not affect the diffusivity, while high levels of disorder creates a percolation network for fast cation diffusion, and increases the probability of antisite annihilation, which in turn reduces the diffusivity.

## Results

### Cation diffusion versus cation disorder

Using traditional and accelerated molecular dynamics (MD and AMD, respectively) simulations, we simulated the diffusion of a Ti vacancy in GTO. For low disorder levels, AMD (i.e., ParSplice, see Methods) simulations are needed in order to achieve longer time-scales over which cation diffusion is active. ParSplice simulations were performed for initial disorder levels between 6.25 and 50% (Table 1). Results show that the Ti vacancy immediately decays into V_Gd_ + Gd_Ti_, as was previously observed in ordered Lu_2_Ti_2_O_7_^27^, such that the resulting initial number of antisites is higher by one than initially constructed. For higher levels of disorder, cation diffusion is significantly enhanced, and we use traditional MD simulations instead. Since these simulations require considerably less computer resources than ParSplice runs, we performed five simulations for each level of disorder, using different structures that are all representative of the material at a given level of disorder.

**Table 1:** Summary of PRD and ParSplice simulations of cation vacancy migration as a function of cation disorder

*N*_anti_ [initial] (disorder)	*N*_anti_ [final] (disorder)	Number of transitions	Simulation time (μs)
0 (0.0%)	0 (0.0%)	360	3.3
9 (7.0%)	9 (7.0%)	1065	4.5
17 (13.3%)	15 (11.7%)	5303	15.2
24 (18.8%)	23 (18.0%)	3549	5.2
39 (30.5%)	29 (22.7%)	5904	3.3
51 (39.8%)	35 (27.3%)	9562	2.8
65 (50.8%)	39 (30.5%)	7977	1.9

Shown are the initial and final number of antisites (and the associated disorder level), the number of transitions (i.e., vacancy moves), and the total simulation time reached. Only the first listed simulation, with disorder *y* = 0%, was performed with PRD

**Fig. 2:**
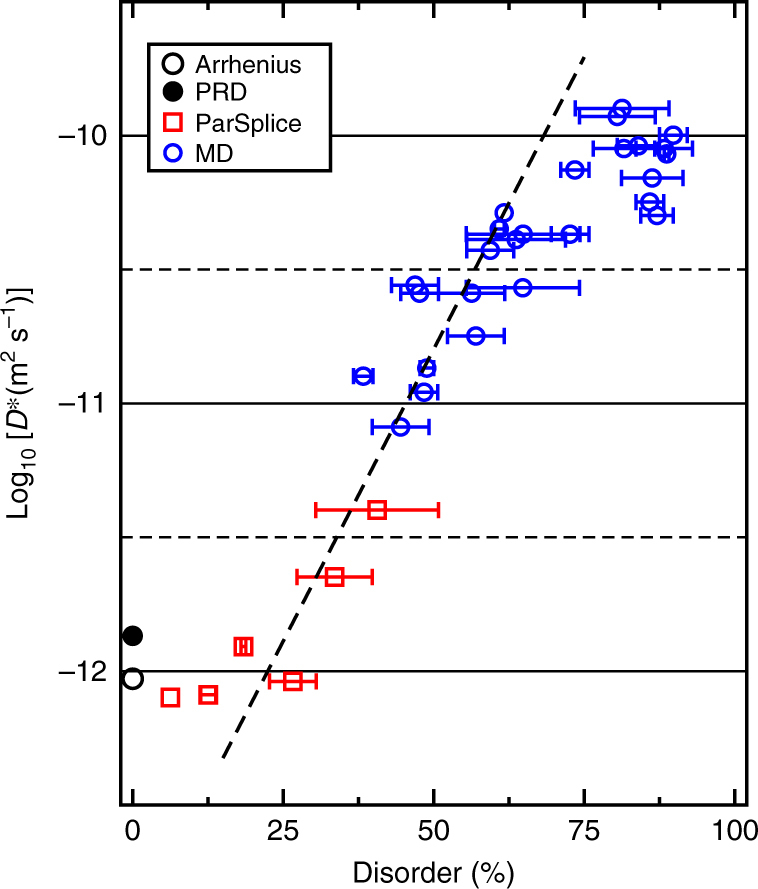
Non-equilibrium diffusion coefficient *D* ^∗^ for cations due to vacancy-mediated diffusion in GTO. See text for the description of *D* ^∗^. Points were obtained using PRD, ParSplice, and MD, as well as predicted from static calculations (see text), and are plotted as a function of cation disorder. The width of the horizontal segments reflect the decrease in disorder observed in the material during the course of the given simulation. The dashed line is a linear fit to the data in the interval *y* = 25–62.5%

Figure 2 summarizes the results obtained from the ParSplice and MD simulations for the cation diffusion due to a cation vacancy in GTO. The diffusion coefficients were extracted from the mean-squared atomic displacements (MSD) averaged over entire simulations. It should be noted that, since the disorder evolves during the simulation (see below), the diffusion constant is not measured in equilibrium conditions. The “non-equilibrium diffusion coefficient” we measure is therefore denoted by *D* ^∗^, to differentiate it from an equilibrium diffusion constant *D*. Note, however, that the change in disorder during a typical simulation is small compared to the change required to induce a large change in the diffusivity. For zero disorder, two additional approaches are employed to determine the diffusion constant. First, as the cation lattice is ordered and thus there is no superbasin (the oxygen ions do not diffuse in ordered GTO), we can use the standard Parallel Replica Dynamics (PRD) method, of which ParSplice is an extension, to simulate cation migration. Second, the diffusion constant can be described by an Arrhenius relation:

1 $${\rm{D}} = {{\rm{D}}_0} \times {\rm{exp}}\left( {\frac{{ - {{\rm{E}}_{\rm{a}}}}}{{{{\rm{k}}_{\rm{B}}}{\rm{T}}}}} \right),$$

where *D*_0_ is the temperature independent prefactor, *E*_a_ the activation energy, *k*_B_ the Boltzmann constant and *T* the temperature. *E*_a_ is obtained by performing nudged elastic band (NEB^34^) calculations for the migration of a Gd vacancy. The prefactor *D*_0_ is:

2 $${{\rm{D}}_0} = \frac{1}{{2{\rm{N}}}} \times {\rm{f}} \times {{\rm{S}}^2} \times {\rm{Z}} \times {\nu _{\rm{m}}},$$

with *N* the dimensionality of the system, *f* the correlation factor (here approximated as 1), *S* the jump distance (3.6 Å), and *Z* the number of neighboring sites the vacancy can hop to (3 of the same chemistry). The attempt frequency *ν*_m_ is obtained via calculation of the normal modes of vibration (Vineyard method^35^):

3 $${\nu _m} = \mathop {\prod}\limits_{n = 1}^{3N - 3} \nu _n^{initial}{\rm{/}}\mathop {\prod}\limits_{n = 1}^{3N - 4} \nu _n^{saddle},$$

with $\nu _n^{\rm saddle}$ and $\nu _n^{\rm initial}$ the normal modes at the saddle and the initial state, respectively. The three translational modes are excluded, as well as the additional imaginary mode characterizing the saddle. In this work, we obtained *E*_a_ = 4.84 eV and ν_m_ = 1.04 × 10^13^ Hz for Gd → V_Gd_ migration.

From Fig. 2, we see that the diffusion constant is insensitive to the disorder level until *y* ~ 25%, after which it rapidly increases (by two orders of magnitude). This increase in diffusivity appears to slow down for disorder level above *y* ~ 50%, but it is difficult to assess whether the diffusivity fully saturates close to full disorder. The horizontal bars on the figure represent the change in disorder during the course of the given simulation. As discussed below, as the vacancy migrates through the material, it can enable the annihilation of antisites and hence promote the reordering of the material. As an aside, we have determined that, despite a variety of local environments, almost all migration events consist of a single atom jumping to the neighboring vacancy, regardless of the level of disorder (Supplementary Note 1). Importantly, the amount of reordering depends on the given simulation, even for the same initial level of disorder. Also, for a given level of disorder, different simulations result in a different value of, and thus a spread in, the diffusion constant. These two observations highlight the effect of the local cation environment on the cation mobility: depending on the local cation arrangement, both the rate of migration and the rate of antisite annihilation can be relatively fast or slow.

### Evolution of cation disorder during diffusion

**Fig. 3:**
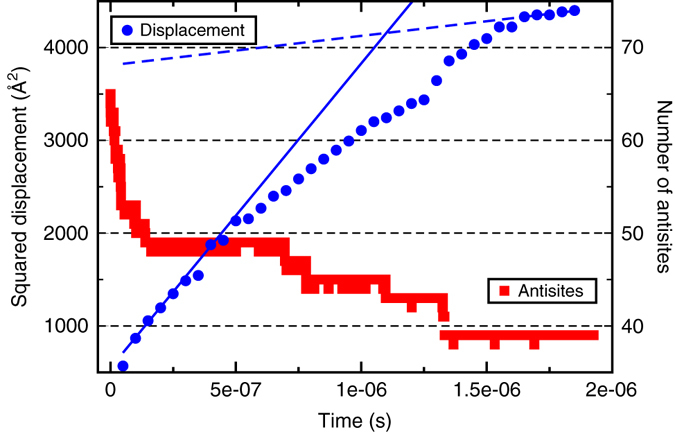
Evolution of the number of antisites and cation squared displacement over time. The plotted data corresponds to a ParSplice simulation of cation vacancy diffusion in a sample with 65 antisites initially. The *solid and dashed lines* indicate the rate of change of the displacement at the beginning and end of the simulation, respectively

To illustrate the dynamical nature of cation diffusion in the disordered material, additional details from a particular case, the ParSplice simulation with starting disorder 50.8%, are shown in Fig. 3. The evolution of the total cation squared displacement shows a trend for the rate of displacement to decrease as the simulation progresses, as indicated by the slopes at the beginning and the end of the simulation. Further, the evolution of the number of antisites shows that it does not steadily decrease. Rather, bursts of fast antitisite annihilation are followed by long periods when the number of antisites remains constant. In addition, the average rate of antisite annihilation seems to decrease as well, with 16 antisites annihilated in the first 0.25 μs, but only 10 in the remaining 1.5 μs. After 1.8 μs, the total number of antisites has decreased by 20%, bringing the disorder level down to 30.5%. Thus, the rate of vacancy diffusion and the rate of antisite annihilation appear to be intimately coupled.

**Fig. 4:**
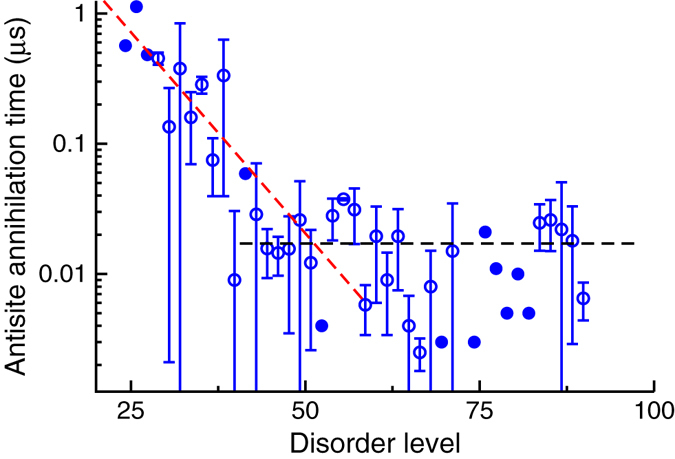
Time to annihilate an antisite, as a function of the number of antisites in the system. Closed symbols denote cases for which only one data point is available, i.e., the transition *n*→*n*−1 antisites was observed only once over all simulations. *Error bars* reflect the standard error for averaged values, when more than one data point was available. The *red dashed line* is obtained by fitting an exponential function, and the *black dashed line* by fitting to a constant

To further illustrate the relationship between the level of disorder and the rate of reordering (antisite annihilation), we analyzed the rate of antisite annihilation as a function of disorder as observed in all the simulations (MD and ParSplice); this data is compiled in Fig. 4. The disorder is obtained by comparing the structure to an ordered reference to identify the number of antisites (this is the lattice reference method); it is worth mentioning, however, that finding the reference corresponding to a fully disordered sample is not trivial. Indeed, the lattice reference method implies that one can clearly identify the ordered and disordered domains of the material, which is not straightforward for a fully disordered system. Here, we shift the reference structure onto all cation sites of the disordered sample, and look for the minimum mismatch (i.e., number of antisites). Over the course of the material evolution however, since the reordering happens at the local level, it may happen that the minimum mismatch configurations differ for the beginning and end of the run; in this case, tracking the disorder evolution is ambiguous and such points were excluded from the data in Fig. 4.

It can be seen from Fig. 4 that the time necessary to annihilate an antisite pair quickly drops with increasing numbers of antisites in the system. There are two main reasons for this: first, a higher vacancy mobility increases the rate at which the vacancy can find and annihilate antisites, and second, a higher concentration of antisites leads to a higher concentration of antisite pairs. This last point is crucial as antisite annihilation occurs through the reaction:

4 $${{\rm V}_{{\rm{Gd}}}}{\rm{ + }}\left( {{\rm{G}}{{\rm{d}}_{{\rm{Ti}}}}{\rm{ + T}}{{\rm{i}}_{{\rm{Gd}}}}} \right) \to {\rm{G}}{{\rm{d}}_{{\rm{Gd}}}} + {{\rm V}_{{\rm{Ti}}}} + {\rm{T}}{{\rm{i}}_{{\rm{Gd}}}} \to {\rm{T}}{{\rm{i}}_{{\rm{Ti}}}}{\rm{ + G}}{{\rm{d}}_{{\rm{Gd}}}}{\rm{ + }}{{\rm V}_{{\rm{Gd}}}}.$$

If opposite antisites are not neighbors, the V_Ti_ in the second step of the reaction in Eq. 4 has to diffuse until it finds the second antisite. However, as mentioned previously, V_Ti_ is not kinetically stable in GTO and cannot diffuse through the material. Thus, if Gd_Ti_ is not a neighbor of Ti_Gd_, the second step will essentially reverse, reverting back to V_Gd_ + Ti_Gd_. The two fitting lines in Fig. 4 illustrate that two regimes of antisite annihilation (slow and fast) appear to exist depending on whether whether the disorder is below or above 50%.

**Fig. 5:**
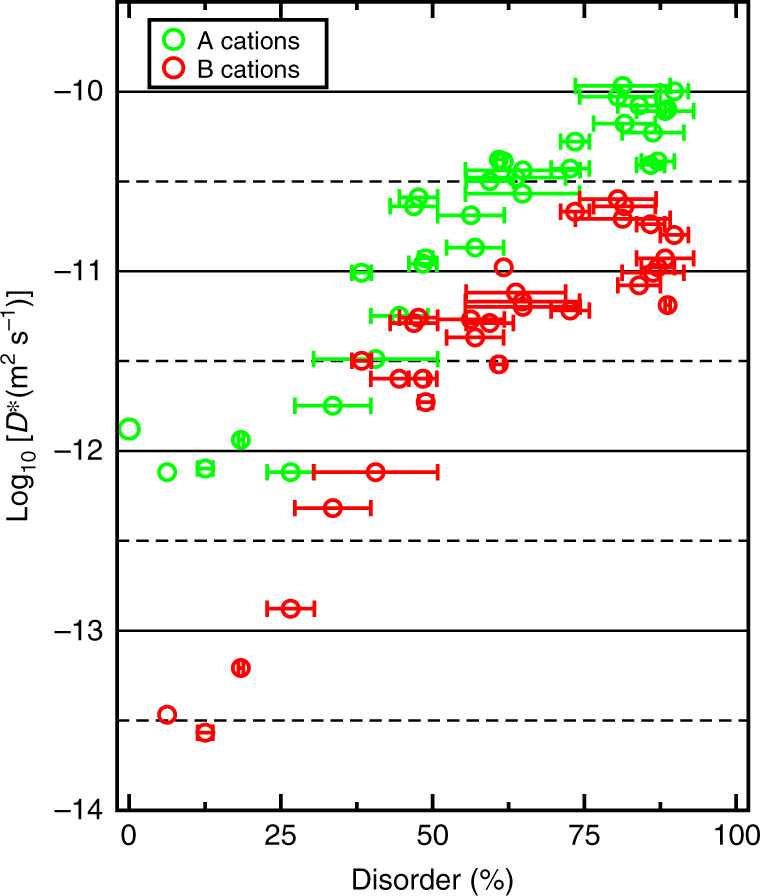
Non-equilibrium diffusion coefficient *D* ^∗^ of A and B cations due to vacancy-mediated diffusion in GTO. See text for the description of D^∗^. Diffusion of A and B cations is shown by *green* and *red symbols*, respectively. Points obtained by PRD, ParSplice, and MD. The width of the horizontal segments reflect the decrease in disorder observed in the material during the course of the given simulation

Note that the diffusion of the vacancy can itself promote the formation of the antisite pairs that are required for ordering. While the fact that the vacancy remains in the V_Gd_ form most of the time limits the diffusivity of the cations on the B lattice, our simulations show that V_Gd_ + Ti_Gd_ ⇔ V_Gd_ + Ti_Gd_ reactions in which V and Ti swap Gd sites are possible, if less probable than V_Gd_ + Gd_Gd_ ⇔ V_Gd_ + Gd_Gd_ reactions. As shown in Fig. 5, in which the individual A and B contributions to the overall cation diffusion are separated, the diffusivity is dominated by A (Gd) cations (also compare with Fig. 2). The diffusivity of B (Ti) cations is initially very low, but steadily increases with disorder, as a result of the process described above. Note that antisite annihilation also contributes to the B diffusivity, especially at higher disorder.

## Discussion

**Fig. 6:**
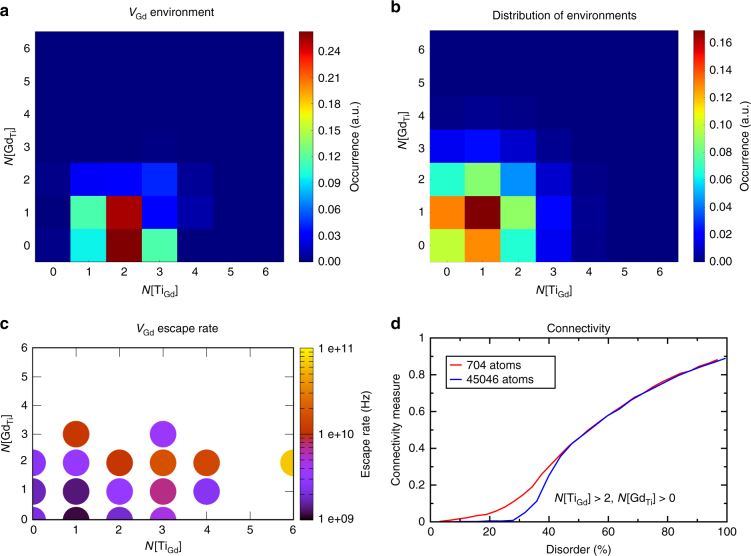
Evidence for percolation diffusion in disordered pyrochlores. **a** Preferred cation environment for the diffusing vacancy during the ParSplice simulation with initial disorder 50.8%. The color reflects the total time spent by the vacancy in a given environment, normalized such that *t*_total_ = 1. **b** Average distribution of antisite environments in a material with disorder *y* = 35%, corresponding to a typical configuration in the simulation in **a**. **c** Average escape rate for the vacancy as a function of local environment for the simulation in **a**. **d** Relative size of the largest connected components of Gd sites with at least 2 Ti antisites neighbors. (corresponding to the site most visited in **a**). The size is normalized by the total number of Gd sites in the material

A key result from our MD and AMD simulations is that there is a threshold level of cation disorder, *y* = 25%, at which there is a sudden increase in the overall cation mobility. This threshold and the shape of the curve in Fig. 2 suggests the occurrence of a percolation network of antisites that facilitates cation vacancy migration above this level of disorder, a hypothesis that is validated in Fig. 6. Several elements are necessary for such an explanation to hold. Firstly, the diffusing species—the cation vacancy—must bind, or have an energetic preference for, the structural elements that define the percolation network. Figure 6a shows the first-nearest-neighbor environment of the Gd vacancy as it migrates during the ParSplice simulation in the sample with initial disorder *y* = 50.8%. The results clearly reveal a preference of the vacancy for environments with 2 Ti antisites and 1 Gd antisite (2,1), and 2 Ti antisites and 0 Gd antisites (2,0). Importantly, this preference is not simply a reflection of the frequency of those environments within the structure.

Indeed, Fig. 6b shows the distribution of cation environments in randomly generated samples at a disorder level of 35% (this particular value of disorder was chosen as representative of the state of the system during most of the simulation, as shown in Fig. 3). Clearly, the preference of V_Gd_ for the (2,1) and (2,0) environments is much higher than their representation in the structure; these sites represent only 15% of all the sites in the material, but the vacancy spent over 50% of the time near them. However, a preference for certain sites is not enough to lead to an enhancement of the diffusivity: there also has to be relatively fast migration between those sites.

Fig. 6c shows, for the same simulation, the dependence of the escape rate of the vacancy on its local environment. Here, it can be seen that the escape rate of the vacancy from a given site increases with the number of antisites around it. In other words, the vacancy quickly diffuses out of sites surrounded by disorder. Combined with the data in Fig. 6a, this means that the vacancy spends more time in sites surrounded by antisites and that the rate at which it moves between these sites is high. Further, the rate to move to other sites surrounded by few antisites has to be low; otherwise the distribution shown in Fig. 6a would not strongly favor antisite-rich environments.

Finally, Fig. 6d displays the relative size of an antisite network as a function of the level of disorder in the material. The curves were generated by analyzing random distributions of antisites for a given disorder level and computing the fraction of A sites that are contained within the largest connected region of first-neighbor A sites with at least 2 Ti_Gd_ neighbors. Two sizes of the system were considered: 704 atoms (the system used in all our simulations), and 45046 atoms (2 × 2 × 2 times the original system), to approach the infinite limit. As can be seen, the relative size of the largest connected clusters quickly increases above *y* = 25%, which is a finite-size signature of percolation (in an infinite system, this measure would jump from 0 to a finite value at the percolation threshold; see, for instance, ref. ^36^). This is the same level of disorder at which we observe cation mobility to start to increase in our simulations (Fig. 2). From the larger system, we determine the percolation threshold to occur around 35–37% disorder.

To summarize our analysis of the percolation behavior in this system: First, the diffusing species (V_Gd_) strongly favors sites surrounded by antisite disorder. Second, the hopping rate between such sites is high. Third, the connectivity of these sites increases quickly above *y* = 25% disorder. Altogether, these observations lead to the conclusion that vacancy-mediated cation diffusion is indeed enhanced by the occurrence of a percolation network of antisites for disorder levels above *y* = 25%.

Remarkably, the existence of a percolation transition in the diffusion of cations is in marked contrast with what is observed for oxygen, where the diffusion steadily increases with disorder^17^. This is likely a consequence of the high number of carriers that dictate oxygen diffusion in disordered pyrochlore, necessitating larger domains of order and disorder to form a percolation network. Indeed, we observed trapping when intrinsic (non-structural) oxygen defects were simulated^17^. Our results are also contrary to observations in spinel^37^ and perovskites^38, 39^, where the oxygen vacancy diffusivity is hindered by cation disorder. While in perovskites, cation ordering was shown to open particular channels for fast diffusion;^39, 40^, the effect of ordering in spinel is less clear. One reason for the different behavior could be that the antisite network was fundamentally different in the simulations from ref. ^37^, either because of the way it was generated (using Monte Carlo in that study versus random generation here) or the crystal structure and stoichiometry of the two materials leads to different relationships between antisite environments. (Indeed, short range order has been observed in ir﻿radiated pyrochlores^58^). Further work is needed to understand these differences. However, it is very clear that cation disorder in complex oxides can lead to very different effects on diffusion, depending on a number of factors.

At this point, it is prudent to discuss the implications of two limitations of our model. First, our simulations were performed at an extremely high temperature (3500 K). While this temperature is much higher than those of interest for this material, we did not observe any melting of either the cation or anion sublattices during our simulations (Supplementary Note 2, and Supplementary Figs. 1, 2). Therefore, we are confident that the observations made in this work are representative of the behavior at lower temperatures of interest (on a much longer timescale of course). Secondly, the concentration of vacancies in our simulation cells is relatively high, especially considering the high formation energy of cation vacancies^41^, due to the small size of the samples. Nonetheless, since we are more interested in providing a physical mechanism relating cation mobility with cation disorder rather than quantitative predictions, the high concentration does not adversely influence our conclusions. That said, the presence of an isolated vacancy does prevent any consideration of vacancy clustering, which may occur in the real material under irradiation conditions^42^ (the concentration of thermal vacancies is not expected to be high enough to allow for clustering). However, a full description of defect interactions in irradiated samples would necessarily involve more complex processes, such as interstitial-vacancy recombination, which are beyond the scope of this paper.

Finally, there is a vast literature on diffusion in disordered media, with various models proposed to describe such systems, from random barrier and random trap models, to models that combine both features (see ref. ^43^ for a brief summary and references therein for more detail). Our results suggest yet another type of model for cation diffusion in disordered pyrochlore. Antisites lead to deeper (trap) states for the vacancy, but critically, also to lower barriers (faster rates) between those sites. Thus, once a percolation network is established, diffusion is enhanced because, as described above, the rate of hopping between antisite environments is faster than between other types of environments. If this faster rate were not present, the formation of a percolation network of traps would not lead to faster diffusion. This effect is similar to that observed for Li diffusion in lithium-transition metal oxides^44, 45^. We note that our percolation threshold is higher than that found by Lorenz and Ziff^46^ for simple fcc lattices: 12% for bond percolation (the study also reports 18% for site percolation, from ref. ^47^). The higher threshold is a consequence of the fact that percolation, in our system, is dictated by the formation of sites with a particular environment (more than one antisite neighbor) that is qualitatively different than the formation of bonds on an fcc lattice with one species. Another important feature of the landscape of cation vacancy diffusion in disordered pyrochlore is the time-dependent nature of that landscape. As time progresses and the cation vacancy reorders the material, traps disappear and the system becomes more homogenous. Further, that antisite annihilation in this system is exothermic naturally leads to lower barriers via the Bell–Evans–Polanyi principle. As discussed above, antisite annihilation is not necessary for high cation mobility in disordered pyrochlore, but it likely contributes to it via the lower barriers associated with annihilation.

A second key result from our study is captured by Fig. 3: cation diffusivity is higher in disordered pyrochlore but that enhanced vacancy diffusivity also drives the system to reorder. This ordering in turn leads to a slowdown of the vacancy diffusion and hence of the recovery rate. Note however that vacancy diffusivity is only one of the contributors to the recovery rate, the others being the concentration of pairs of neighboring antisites and a possible dependence of the rate of the V_Ti_ + Ti_Gd_ → V_Gd_ reaction on the local environment. These additional factors could explain why the cation diffusivity and the recovery rate do not abruptly increase at the same level of disorder.

To conclude, we used molecular dynamics and ParSplice simulations to investigate cation vacancy diffusion in disordered Gd_2_Ti_2_O_7_ pyrochlore. We find that the diffusion in materials with low disorder is slow, independent of disorder, and almost exclusively comprised of Gd cation diffusion. However, above a threshold of *y* = 25% disorder, the diffusivity increases sharply, along with the contribution of Ti cations. We find evidence that the fast diffusion is due to the formation of a percolation network of antisites, in which the vacancy can diffuse quickly. Finally, we find that the vacancy can heal the disorder in the material, at a rate that increases with the amount of disorder in the material. The diffusion of cations in GTO is thus a complex phenomena, where cation disorder allows for fast diffusion in a percolation network above a certain level and thus enhances healing of the material, which in turn reduces the diffusivity and the healing rate. Together, these results highlight the complex interplay between cation diffusion and disorder in pyrochlores, and provide insights into sintering, radiation damage, and aging processes.

## Methods

### Molecular dynamics and disorder measure

The atomic interactions of the Gd–Ti–O system are modeled by the Buckingham potential:

5 $$V\left( r \right) = {{A}} \times {\rm{exp}}\left( { - \frac{r}{\rho }} \right) - \frac{{\rm{C}}}{{{r^6}}},$$

where *A*, *C*, and *ρ* are adjustable parameters. Formal charges are adopted, and the Coulomb interaction is calculated via the Ewald sum technique. The parameterization of Minervini et al.^48^ was adopted, as shown in Table 2. Although the Buckingham potential form is rather simple, and notably does not allow for charge transfer between ions, it has been widely used to study the properties of complex oxides, and has been repeatedly demonstrated to reveal physically meaningful trends in these types of materials, in agreement with experimental^7, 8^ and density functional theory^49, 50^ results.

**Table 2:** Parameterization of the Buckingham potential used in this study

Interaction	*A* (eV)	*ρ* (Å)	*C* (eV Å^−6^)
O^2−^–O^2−^	9547.96	0.2192	32.0
Gd^3+^–O^2−^	1885.75	0.3399	20.34
Ti^4+^–O^2−^	2131.04	0.3038	0.0

All parameters from ref. ^48^

The MD simulations are performed with the LAMMPS code^51^, in the NPT ensemble at zero pressure and 3500 K. This temperature is significantly higher than the physical conditions of interest, although it is low enough to prevent melting of the samples, and the temperature simply speeds up the cation transitions (Supplementary Note 2). Each sample consists of 2 × 2 × 2 unit cells of pyrochlore (~20 × 20 × 20 Å^3^, 704 atoms), in which A and B cations are randomly swapped to create the desired level of disorder. The resulting samples, with chemical formula (Gd_1−*x*_Ti_*x*_)_2_(Gd_*x*_Ti_1−*x*_)_2_O_7_, with 0 < *x* < 0.5, have a percent disorder level of:

6 $$y = 2 \times x \times 100.$$

In a fully disordered case (100%), the occupancy on each cation crystallographic site is half A and half B, and the disorder is maximal (2 × 0.5 × 100 = 100%). This measure of disorder via site mixing is similar to that employed in experimental studies^52^. In this paper, the level of disorder is measured through the so-called reference lattice method^53^, whereby antisites are identified through comparison with a reference perfect lattice. As shown in the Supplementary Note 3 and Supplementary Fig. 3, our conclusions also hold when a different measure of disorder, one which would correctly quantify disorder in samples that contain ordered domains, is used instead. Before the simulations begin, a Ti cation vacancy is introduced in the sample, which is then equilibrated at the desired temperature by running for 10000 steps (10 ps).

### ParSplice

As was shown above, disorder promotes fast cation diffusion so that direct MD simulations are adequate at high disorder; cation diffusion is however considerably slower at low disorder. In this regime, we instead rely on the parallel trajectory splicing^32^ method. ParSplice is an extension of parallel replica dynamics (PRD^54, 55^). In PRD, the simulation is parallelized in the time domain by simultaneously exploring a given state with multiple replicas. However, in the standard PRD method, states are visited sequentially. Therefore, the efficiency of a PRD simulation, and thus the number of processors that can be applied, is limited by the escape time out of individual states. ParSplice overcomes this limitation by exploring multiple states at once. If the state-to-state trajectory enters one of these states, the pre-calculated segments of trajectory can be directly spliced onto the existing one. Segments are carried out in different states according to the estimated likelihood that they will be visited by the trajectory in the future; this likelihood is obtained from a kinetic model that is updated on the fly as the simulation proceeds. Note that speculation with regard to the states that will be visited in the future only serves to allocate “excess” resources that could not be efficiently leveraged by a traditional PRD approach; ParSplice therefore always outperforms PRD, especially so for systems containing sets of states that are revisited many times before the trajectory moves on to a different region of configuration space (so-called “superbasins”)^56^.

In the ParSplice simulations, states are defined based on the position of cations alone, i.e., a state-to-state transition is deemed to have occurred only when there is a change on the cation lattice. This is justified by the fact that fast oxygen motion allows for a rapid equilibration with respect to the instantaneous cation configuration. Computational studies on La_2_Zr_2_O_7_ notably showed that the oxygen sublattice quickly (0.5 ps) responds to an imposed cation disorder^57^, a result that should apply for other pyrochlore chemistries. This was explicitly verified here by demonstrating that the waiting times for a cation jump event is an exponentially distributed random variable, indicating a sufficient separation of timescales between cation and oxygen dynamics. Details are given in the Supplementary Note 4 and Supplementary Fig. 4.

### Data availability

The data that support the findings of this study are available from the corresponding authors upon reasonable request.

## Electronic supplementary material

Supplementary Information
