# Supplementary material for: Evidence for percolation diffusion of cations and reordering in disordered pyrochlore from accelerated molecular dynamics
Source: Nat Commun. 2017 Sep 20;8:618. doi: 10.1038/s41467-017-00708-z (PMC5606988; doi:10.1038/s41467-017-00708-z)
Supplement: Supplementary file 1 — Supplementary Information [file 41467_2017_708_MOESM1_ESM.pdf]

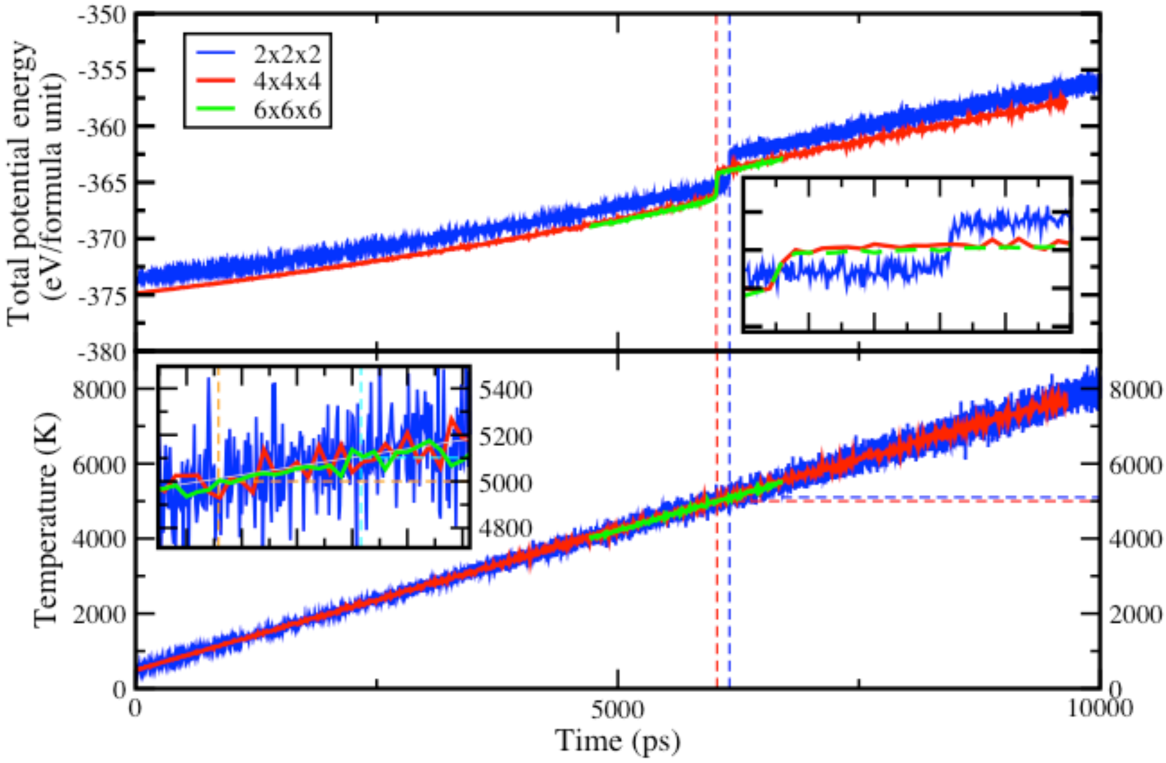

Supplementary Figure 1: Total potential energy and temperature during NPT simulations of disordered GTO+VTi melting, for three system sizes. Inserts show details used to determine the melting temperature.

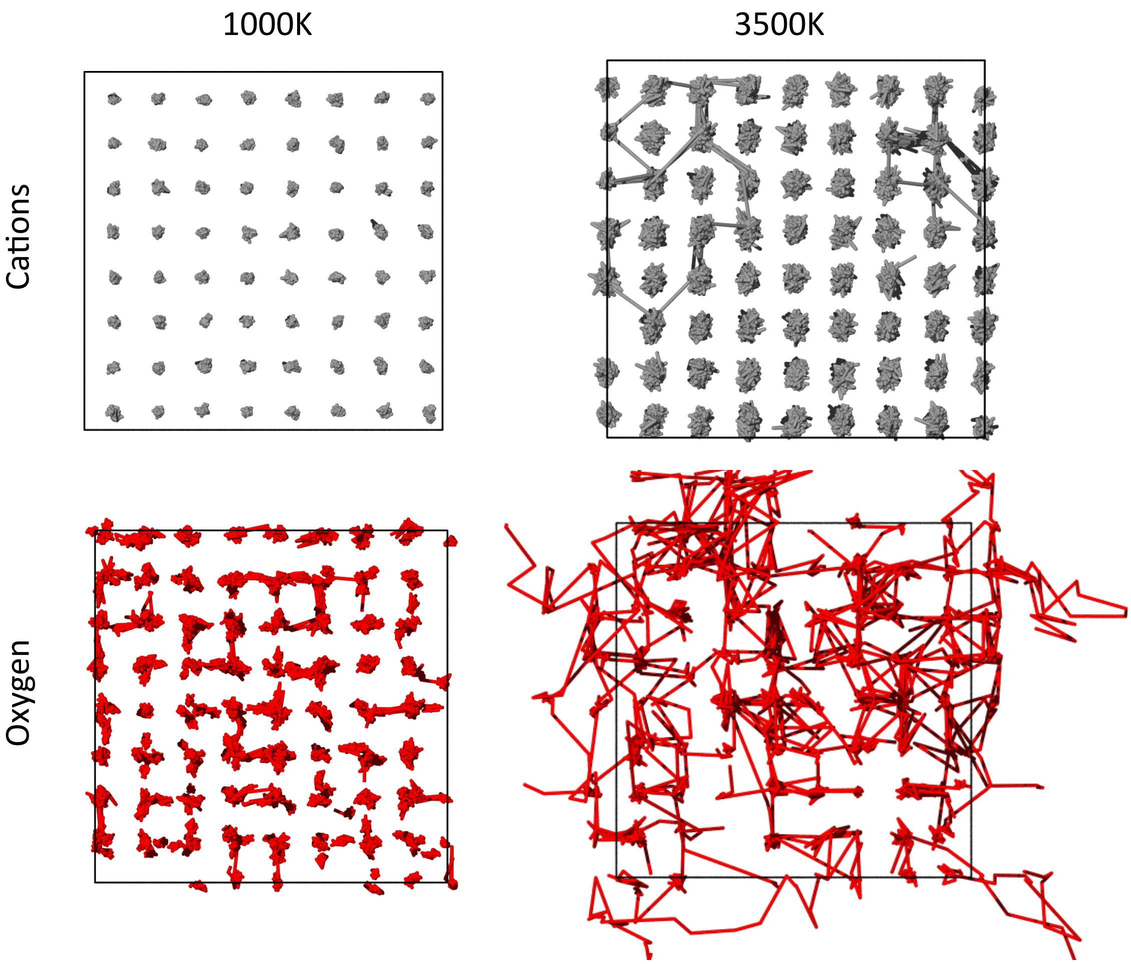

Supplementary Figure 2: Cation and oxygen trajectories in a disordered GTO sample with a cation vacancy, over 0.5 ns with a time resolution of 10 ps. Left: T=1000K; Right: T=3500K. For better visibility, only oxygens belonging do a thin slice of width 5 A are shown at 3500K, and over a reduced time (0.25 ns).

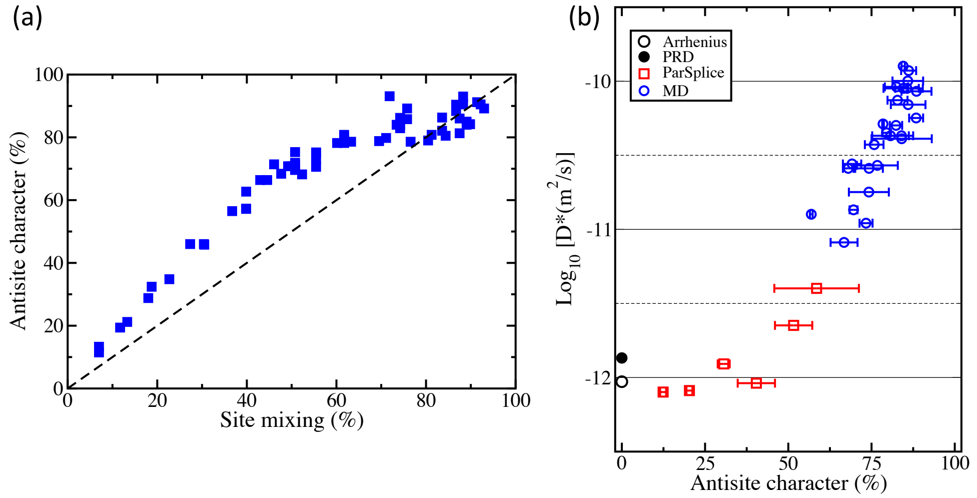

Supplementary Figure 3: Comparison between the antisite character and site-mixing measures of disorder in the material (a), and Non-equilibrium diffusion coefficient D* (see main manuscript) for cations as a function of the antisite character in the material with a cation vacancy (b)

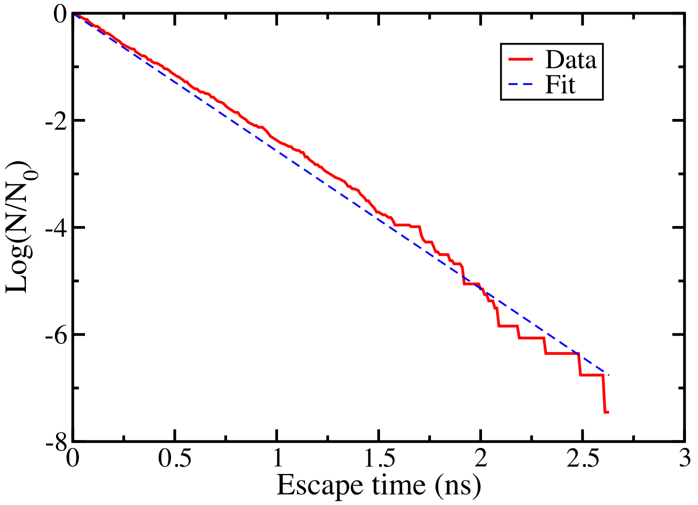

Supplementary Figure 4: Time for a cation vacancy in GTO to hop, or escape from its current state, at T=3000 K. N_0 is the total number of simulations (1724) and N is the number of simulations in which a cation jump has not been observed. The system exhibits exponential behavior (straight line fit in the log scale plot), characteristic of first order-transition behavior.

Supplementary Note 1:
Characterization of migration events

From the ParSplice simulations, we have analyzed the frames for the runs with 6.25 and 12.5% of disorder initially. Frame-to-frame comparison revealed that all but a few events (<1%) consist of a single atomic jump, to a nearest-neighbor position.

From the MD simulations, atomic displacements larger than 2 Angstrom were identified with the available time resolution (10 ps). For the samples with 50, 75, and 100% disorder initially, single events form 92, 90, and 87% of the total numbers of events, respectively. It should be noted that this estimation is actually a lower bound, since all multiple events occurring during the 10 ps time window count as concerted moves, even though they might have occurred independently.

Supplementary Note 2:
High temperature behavior

The melting temperature of GTO pyrochlore was evaluated by performing NPT simulations on a disordered system with a Ti vacancy. The simulation consists of increasing the temperature from 500K to 8000K, at zero pressure, over a total time of 10 ns (10^7 steps). The results are shown in Supplementary Figure 1, for systems with size 2x2x2 (703 atoms, same as the one used in the main manuscript), 4x4x4 (5631 atoms) and 6x6x6 (19007 atoms). Supplementary Figure 1 shows that the sample completely melts at 5100 K for the 2x2x2, and 5000 K for the larger sizes. While the ramping method is less accurate than monitoring the evolution of the liquid/solid interface at fixed temperature, it allows for a quick estimation of the melting temperature, and the slow ramping limits overestimation. A simulation made from 4000 to 5500 K over the same time (i.e. 150 K/ns, instead of 750K/ns, temperature increase rate) yielded a melting temperature of 4800 K, still much higher than T=3500 K used in the main simulations. By the same method as described above, the melting point of the ordered system with a Ti vacancy is found to be much higher, around 7000 K.

We note that this melting temperature is much higher than the real material, which is closer to 1820 C. The extremely high temperature for the melting temperature found with the potential is at least partially a consequence of our not using shells and partially a simple failure of this potential. That said, these potentials have been seen to give reasonable values for migration barriers in a number of scenarios, so we expect them to give a reasonable description of defect migration. The failure to describe the melting temperature is likely a consequence of an inadequate description of the Frenkel pair formation energy; these types of potentials are known to over-estimate the formation energy of defects. Thus, while we do not expect a good description of the melting temperature, we expect that the qualitative kinetic behavior we are describing in this paper is reasonable.

In agreement with the determined melting temperature, Supplementary Figure 2 displays the trajectory of cations and anions over a time interval of 50 ps, at 1000 K and 3500 K. The crystalline nature of the system, even at high temperature, is obvious, with cations and oxygens clearly attached to a particular site before possible migration.

Supplementary Note 3:
Effect of the disorder measure

As discussed above, the level of disorder is measured through the so-called reference lattice method [1], whereby antisites are identified through comparison with a reference perfect lattice. This provides a measure of site mixing relative to a reference lattice. However, one could imagine the nucleation of spatially distinct regions of ordered pyrochlore, in which case the resulting material would be composed of ordered pyrochlore domains shifted relative to one another and separated by domain boundaries. Even though such a case is unlikely in the small unit cells used here, this reference lattice method would lead to an incorrect estimation of the material's disorder, as the comparison to a reference structure could only recognize one domain as pyrochlore, while the other domains would appear as fully disordered. For this reason, a second disorder measure is employed, which identifies the “antisite character” of the material, by looking at the second nearest-neighbor environment of each atom. In perfect pyrochlore, the six second nearest-neighbors of a given cation are cations of the opposite species, while in a fully disordered material (*i.e.* defect fluorite) the environment would be equally mixed (50% A/B occupancy on each cation site). Thus, the antisite character is defined as:

$$\chi = \frac{n_{average}}{n_{fluorite}} * 100, \qquad (1)$$

where $n_{average}$ is the average number of cations of the same species in the second nearest-neighbor shell, and $n_{fluorite}=3$. A similar measure was used in Ref. [2] to define a cation order parameter. The antisite character (Eq. (1)), being a local measure does not depend on any reference, ensures that shifted pyrochlore domains would indeed appear to form a mostly ordered material, apart from the boundary regions.

The comparison between the two measures is presented in Supplementary Figure 3-a. It can be seen that the two measures agree at the high end of the disorder, but that the antisite character is consistently larger than the site mixing at medium and low site mixing. This is because the nature of the second nearest-neighbor environment differs for isolated antisites -- typical of low to moderate disorder and having only cations of the same species as second nearest-neighbors - and defect fluorite - which has a fully mixed occupancy. In addition, the antisite character is less sensitive to changes in site mixing at higher levels of site mixing.
In Supplementary Figure 3-b, the diffusivity *vs.* disorder results shown in the main manuscript in Fig. 1 are replotted with respect to the antisite character instead of the site mixing, in order to further validate the conclusions made earlier. It can be seen that the curve has a similar shape, only shifted to the right: below $\chi \approx 50\%$ antisite character, slow

and constant cation diffusion is observed. Above, a sharp enhancement is observed up to the maximum levels of antisite character. Further, the measured changes in disorder during the course of the various simulations is similar between the two methods, though the measure $\chi \approx 50\%$ of antisite character changes less at high disorder than does the site mixing measure, a consequence of the relation exposed in Supplementary Figure 3-a. The shift of the threshold for high diffusivity is also rooted in this relation site mixing - antisite character.

Supplementary Note 4:
State definition in ParSplice

One of the challenges in simulating the long-time behavior of this material is the fact that anions and cations diffuse at very different rates. In order to address this issue, we defined states in ParSplice in terms of the cations alone; *i.e.*, transitions are only deemed to have occurred if the cation lattice reorganizes; specifically, a transition is detected when the occupation of the Voronoi volumes defined on the perfect lattice changes. This definition of the states according to the cations yields accurate dynamics and efficient simulations as long as anions can be considered to rapidly achieve local equilibrium with respect to the instantaneous configuration of the cations [3]. In this limit, the state-to-state dynamics should be described by first-order kinetics, and hence the distribution of escape times should be exponential [3]. We verified that this condition is obeyed by running numerous independent simulations (1724 in the present case) and recording for each simulation the time at which a cation jumped to the nearby vacancy. The same initial structure with disorder level 12.5% was used in all the simulations; however, the random seeds for the Langevin thermostat and the initial velocity assignment were randomized, creating independent trajectories. As shown in Supplementary Figure 4, the results indeed indicate that state definition in terms of cations alone is warranted.

Supplementary References:

[1] Uberuaga, B. P.; Choudhury, S.; Caro, A. Ideal Sinks are not Always Ideal: Radiation Damage Accumulation in Nanocomposites. J. Nucl. Mater. 2014, 462, 402–408.

[2] Chartier, A.; Meis, C.; Crocombette, J.; Weber, W. J.; Corrales, L. R. Molecular Dynamic Simulation of Disorder Induced Amorphization in Pyrochlore. Phys. Rev. Lett. 2005, 94, 025505.

[3] Perez, D.; Vogel, T.; Uberuaga, B. P. Diffusion and Transformation Kinetics of Small Helium Clusters in Bulk Tungsten. Phys. Rev. B 2014, 90, 014102.
